# RNA Sequencing of Trigeminal Ganglia in *Rattus Norvegicus* after Glyceryl Trinitrate Infusion with Relevance to Migraine

**DOI:** 10.1371/journal.pone.0155039

**Published:** 2016-05-23

**Authors:** Sara Hougaard Pedersen, Lasse Maretty, Roshni Ramachandran, Jonas Andreas Sibbesen, Victor Yakimov, Rikke Elgaard-Christensen, Thomas Folkmann Hansen, Anders Krogh, Jes Olesen, Inger Jansen-Olesen

**Affiliations:** 1 Danish Headache Center, Department of Neurology, Glostrup Research Institute, Rigshospitalet, and Faculty of Health and Medical Sciences, University of Copenhagen, Copenhagen, Denmark; 2 The Bioinformatics Centre, Department of Biology, University of Copenhagen, Copenhagen, Denmark; International Centre for Genetic Engineering and Biotechnology, ITALY

## Abstract

**Introduction:**

Infusion of glyceryl trinitrate (GTN), a donor of nitric oxide, induces immediate headache in humans that in migraineurs is followed by a delayed migraine attack. In order to achieve increased knowledge of mechanisms activated during GTN-infusion this present study aims to investigate transcriptional responses to GTN-infusion in the rat trigeminal ganglia.

**Methods:**

Rats were infused with GTN or vehicle and trigeminal ganglia were isolated either 30 or 90 minutes post infusion. RNA sequencing was used to investigate transcriptomic changes in response to the treatment. Furthermore, we developed a novel method for Gene Set Analysis Of Variance (GSANOVA) to identify gene sets associated with transcriptional changes across time.

**Results:**

15 genes displayed significant changes in transcription levels in response to GTN-infusion. Ten of these genes showed either sustained up- or down-regulation in the 90-minute period after infusion. The GSANOVA analysis demonstrate enrichment of pathways pointing towards an increase in immune response, signal transduction, and neuroplasticity in response to GTN-infusion. Future functional in-depth studies of these mechanisms are expected to increase our understanding of migraine pathogenesis.

## Introduction

Migraine is a complex disorder involving episodic activation of the trigeminovascular system where the trigeminal ganglia play a pivotal role in initiating and maintaining activation of signal transmission resulting in pain perception [1]. Infusion of some naturally occurring signaling substances induce headache but not pain in the rest of the body [2–4]. The nitric oxide (NO) donor glyceryl trinitrate (GTN), induce an immediate headache that four to six hours later is followed by migraine or cluster headache attacks in respective patients similar to their spontaneous attacks [3,5,6]. The involvement of endogenous NO in migraine pathophysiology was confirmed by the anti-migraine effect of the nitric oxide synthase (NOS) inhibitor L-N^G^-monomethyl arginine citrate (L-NMMA) [7,8].

We have previously developed an experimental animal model where GTN, in a dose equivalent to the human dose, is infused into freely moving rats [9]. This model avoids factors like anesthesia, stress, and therefore closely mirrors the conditions in the human model. In this rat model we observed significantly elevated levels of Fos mRNA at 30 minutes and neurons positive for c-fos protein at four hours after GTN-infusion in the trigeminal nucleus caudalis, indicating neuronal activation in the trigeminal system [9]. The dura mater is innervated by trigeminal neurons and in response to GTN-infusion, nerve fibers containing neuronal NOS increased [10,11]. Infusion of GTN also caused dural mast cell degranulation initiating neurogenic inflammation [12,13]. Pre-treatment with the effective anti-migraine drugs sumatriptan and L-NAME aborted these effects [9,11–13].

Recently, Perkins *et al*. proposed the use of RNA sequencing (RNA-seq) for transcriptomic pain studies instead of traditional microarrays, permitting analysis of an even larger number of putative candidate pain genes as well as for detecting a wider range of expression values [14]. We performed analysis of NO-provoked transcriptional changes by RNA-seq of the complete trigeminal ganglion including all cell types present at two different time points after GTN-infusion. We hypothesize that the identification of transcriptional differences in the trigeminal ganglion between vehicle- and GTN-infused rats will lead to an improved understanding of the molecular mechanisms involved in primary headaches and contribute significantly to the knowledge of migraine pathophysiology.

## Methods

### 2.1 Animals

A total of 12 male *Rattus Norvegicus*, (Sprague-Dawley) weighing 320–340 g (Taconic M&B, Denmark) at the time of infusion were used. The rats were maintained in cages with a 12 hours light/dark cycle and provided with ad libitum access to a standard rodent diet and water. Following surgery, the rats were single housed for nine days. All animal care and experimental procedures complied with institutional guidelines and were approved by the Danish Animal Experiments Inspectorate (License number 2012-15-2934-00697).

### 2.2 Surgical procedure, GTN infusion, and tissue sampling

Cannulation of the femoral vein of 12 male Sprague-Dawley rats was performed as previously described (9). The catheter was placed SC and exteriorized at the nape of the neck. The rats recovered for a period of seven days and were then transferred to Accusampler cages (Dilab, Lund, Sweden) where the catheter was connected through a tether. In this set-up, they acclimatized for another two days while they were able to move freely within the cage. On day nine post-operatively, the animals were infused with a low dose of GTN, allometrically translated from the human dose, of 4 μg/kg/min, infused over 20 minutes. GTN was diluted in saline from a stock of 5 mg/ml in 96% ethanol (Nycomed, Roskilde, Denmark) and was freshly prepared before each experiment. Based on previous observations of mRNA levels of Fos expression showing equal levels of expression at 30 min and a non-significant trend of increased expression at 1 and 2 hours we choose the 30 and 90 min time points in the present study [9]. The rats were divided into three groups and infused with either 1) vehicle (1.2% ethanol in saline) and sacrificed 30 minutes after the infusion (n = 4), 2) GTN and sacrificed 30 minutes after the infusion (n = 4), or 3) GTN and sacrificed 90 minutes after the infusion (n = 4). At the time of sacrifice, the rats were deeply anesthetized with pentobarbital (Glostrup Hospital Pharmacy, Denmark) and euthanized by transcardial perfusion with ice-cold phosphate buffered saline (PBS). A craniotomy was made and the trigeminal ganglia were rapidly dissected, snap-frozen, and stored at -80°C for RNA extraction. The animals were randomized between experimental groups.

### 2.3 RNA-seq

Total RNA was extracted from the trigeminal ganglion according to manufacturer's protocol using the Isolation of Small and Large RNA-Kit (Macherey-Nagel, Germany) in combination with tissue homogenization with ceramic beads (MO BIO, Carlsbad, USA) and TRIzol® (Invitrogen, USA). RNA quality was assessed using the Bioanalyzer RNA 6000 Nano Kit (Agilent Technologies, CA, USA) and samples with a RNA integrity number (RIN) < 7 were discarded. Based on this criterion two control samples were excluded. The RNA samples were further processed and sequenced by AROS Applied Biotechnology A/S, Aarhus, Denmark. Briefly, the mRNA was isolated using poly(A) selection from 0.4 μg total RNA. The mRNA was chemically fragmented and converted into single stranded cDNA using SuperScript II and random primers. The second strand was synthesized followed by repair of the cDNA ends. A single adenosine was added to the 3’ ends of the double stranded cDNA. Then special Illumina adaptors were ligated onto the cDNA pools using T4 DNA ligase, these included sample-specific barcodes. 100 bp fragments were amplified by 15 cycles PCR. Libraries were validated with an Agilent Bioanalyzer, diluted, and the samples were randomized before they were applied into two lanes on an Illumina flow cell using the Illumina cBOT. Sequencing was performed using the Illumina HiSeq 2000 platform. A total of 500 million paired-end, un-stranded 100 nucleotide reads was generated. The raw sequencing data are available from the NCBI Sequence Read Archive under accession number SRP072027.

### 2.4 Data analysis

#### 2.4.1 Differential expression analysis

Reads were aligned to the *R*. *Norvegicus* genome (Ensembl rn5) using TopHat2 (v2.0.10 run on top of bowtie v2.1.0) provided with the Ensembl transcript annotation (release 75, February 2014) as input and run using “—b2-very-sensitive” and “—library-type fr-unstranded”. The number of uniquely mapped reads falling within each Ensembl gene was then counted using htseq-count (v0.5.4p5, [15]) in un-stranded mode to provide a table with sample read counts for each gene (S1 Dataset).

Differential expression analysis was conducted in R [16] using the DESeq2 package [17]. First, to identify potential outliers hierarchical clustering was performed on the count data after transformation with regularized logarithm. Next, a negative binomial generalized linear model with a dummy variable for each of the two treatment regimens (GTN-30 and GTN-90) was fitted using DESeq2; we note that this implicitly assumes that any vehicle effect was unchanged between the two time points of sacrifice as is the case in previous studies of molecular changes in this model [11,13]. The estimates used for hypothesis testing were obtained without coefficient shrinkage, whereas the estimates used for visualization were shrunk using a zero-centered normal prior. Testing for differential expression in response to GTN-administration was conducted using the likelihood-ratio test comparing the full model with two dummy variables with an intercept-only model. The false discovery rate method was used to adjust for multiple testing.

#### 2.4.2 Gene Set Analysis of Variance (GSANOVA)

A new method named Gene Set Analysis of Variance (GSANOVA) was developed to test for sets of genes that are enriched for changes in expression in response to treatment across the two time-points. Importantly, this improves standard methods like Gene Set Enrichment Analysis (GSEA) by being able to integrate information across time. In essence, the method is a modified version of the Gene Set Analysis (GSA) algorithm [18], where the f-statistic (the ratio of between-group-variation to within-group-variation), rather than the t-statistic, is used as the gene-level statistic to allow for more than two groups (time points in this case). We note that the f-statistic has also previously been proposed in the context of gene set enrichment tests [19].

Our method takes the RNA-seq gene counts for each sample and a set of gene-sets (e.g. gene ontology annotations) as input. First, the regularized logarithmic transformation of the count data is computed using DESeq2 and used as gene expression estimates for the downstream analysis. Then the f-statistic is computed for each gene in the gene-set in question using following equation;

$$f\;=\frac{n-k}{k-1}\frac{\sum_{i=1}^{k}\;n_{i}{\left(\overset{¯}{x}\;-\;\overset{¯}{x}\right)}^{2}}{\sum_{i=1}^{k}\sum_{j=1}^{n_{i}}\;{\left(x_{ij}-{\overset{¯}{x}}_{i}\right)}^{2}}$$

where *k* denotes the number of groups, and *n*_*i*_ and ${\bar{x}}_{i}$ denote the size and mean expression level of group *i* respectively, and *n* and $\bar{x}$ denote the number of individuals and the global mean, respectively. The f-statistic for unexpressed genes is set to zero. The mean f-statistic for the genes in a given gene-set is then used as the raw enrichment score for that gene-set. This score is standardized using the mean and standard deviation of the scores from 1,000 randomly generated gene-sets of the same size. Finally, standardized enrichment scores are calculated for 1,000 random permutations of the group labels and used to compute a *nominal* p-value for each gene-set. To adjust for multiple testing, another set of p-values is first computed using the global null distribution obtained by combining permutation scores across gene-sets [20]; these p-values are then used to compute q-values using the qvalue package in R [21]. Similar to the standard GSEA method, we used a q-value cutoff of 0.25 to select gene sets of interest [20].

### 2.5 Semi-quantitative Reverse Transcriptase-PCR (qPCR)

For validation of the RNA-seq results, genes were selected for qPCR analysis amongst the significantly differentially identified genes. cDNA was synthesized from 1 μg RNA using the iScript cDNA Synthesis Kit (Bio-Rad, CA, USA) according to the manufacturer's protocol. qPCR was performed using the QuantiTect SYBR Green PCR kit (Qiagen, Hilden Germany) according to manufacturer's instructions. Based on a literature search of suitable reference genes in nervous tissue, we screened all samples for several normalization genes and found hypoxanthine phosphoribosyl transferase 1 (Hprt1, QT00365722) to be most stably expressed across the samples [22]. Thus, the expression of target genes was normalized to Hprt1. The following target genes were selected based on RNA-seq data to represent different patterns of regulation: RT1 class I, locus A3 (*RT1-A3*, forward; 5'-CTTCTGTTGTTCTTGGAGCTGTG-3', reverse; 5'-CCACTCCAGGCAGCTGTCTTC-3'), period circadian clock gene 1 (*Per1*, QT00389459), TAP binding protein (Tapasin, *Tapbp*, QT00189819), and regulator of G-protein signaling 7 binding protein (*Rgs7bp*, QT00399105). Primers were either from QuantiTect Primer Assays (Qiagen) or designed due to high sequence homology within the gene family. Primer efficiencies (E) were determined based on previous amplification efficiencies on trigeminal ganglion RNA. Ct denotes threshold cycle and gene expression was calculated using the formula:

$$\mathrm{Relative copynumber}\;={\left(1+\frac{E}{100}\right)}^{-\mathrm{Ct value}}$$

## Results

### 3.1 Differential gene expression

**Fig 1:**
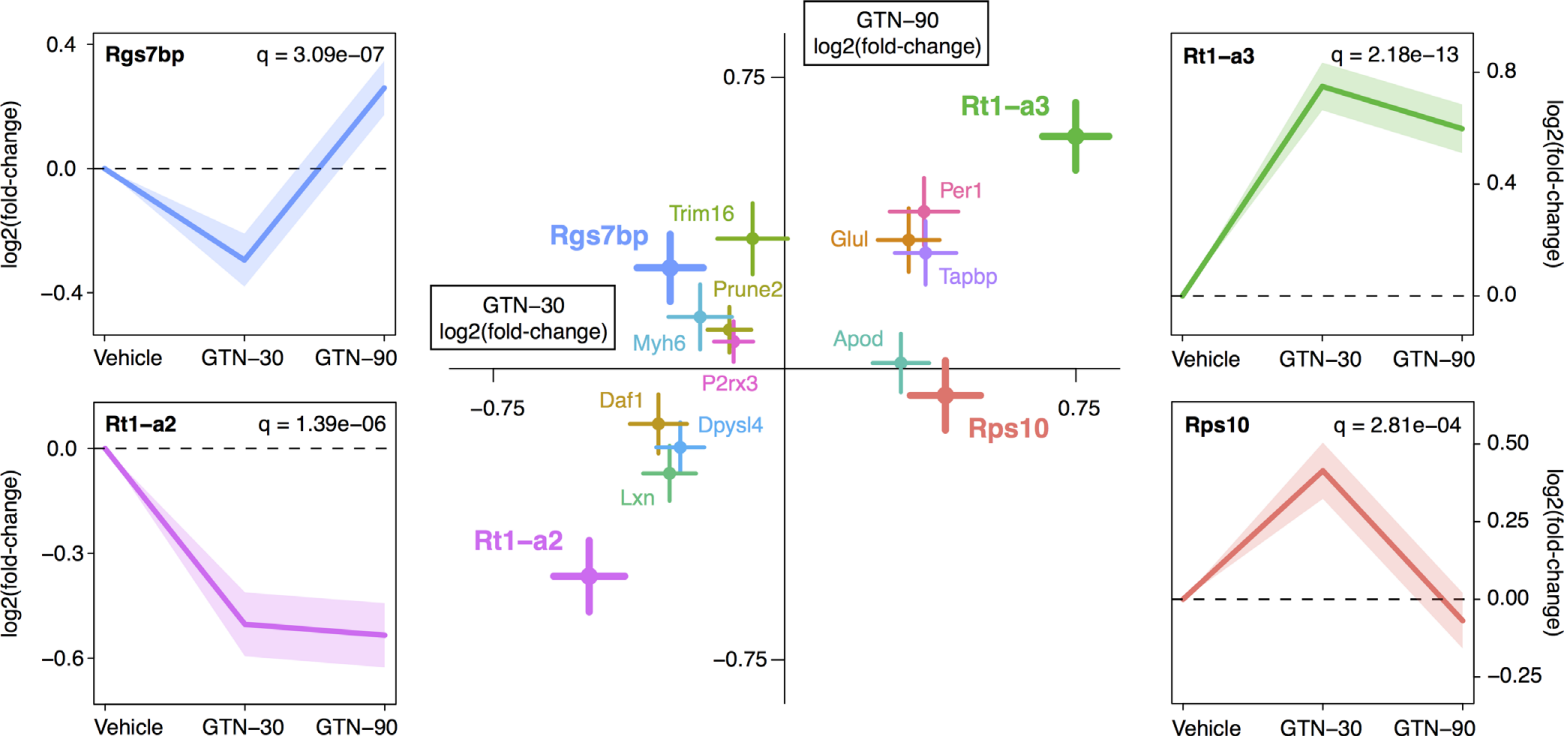
Trigeminal ganglia gene expression dynamics in response to GTN treatment. Differential gene expression analysis was conducted in DESeq2 using likelihood-ratio tests with correction for multiple testing using the False Discovery Rate method. 15 genes exhibited statistically significant changes in gene expression at a false discovery rate cutoff of 5%. In the central plot, dots represent estimated log2(fold-changes) with the X- and Y-dimensions representing the responses at 30 and 90 minutes, respectively. The horizontal and vertical lines show the standard error of the log2(fold changes) after 30 and 90 minutes, respectively. The four lateral line-plots provide an alternative visualization of the gene expression trajectories for the four most significantly regulated genes (RT1-A3, Rps10, RT1-A2 and Rgs7bp).

A single suspected outlier was identified based on hierarchical clustering analysis and was excluded from further analysis (S1 Fig). Differential gene expression analysis was conducted on the remaining samples using DESeq2. Fifteen genes were significantly (q<0.05) differentially expressed in response to GTN infusion as compared with vehicle treated animals (Fig 1, S1 Table and S2 Fig).

**Fig 2:**
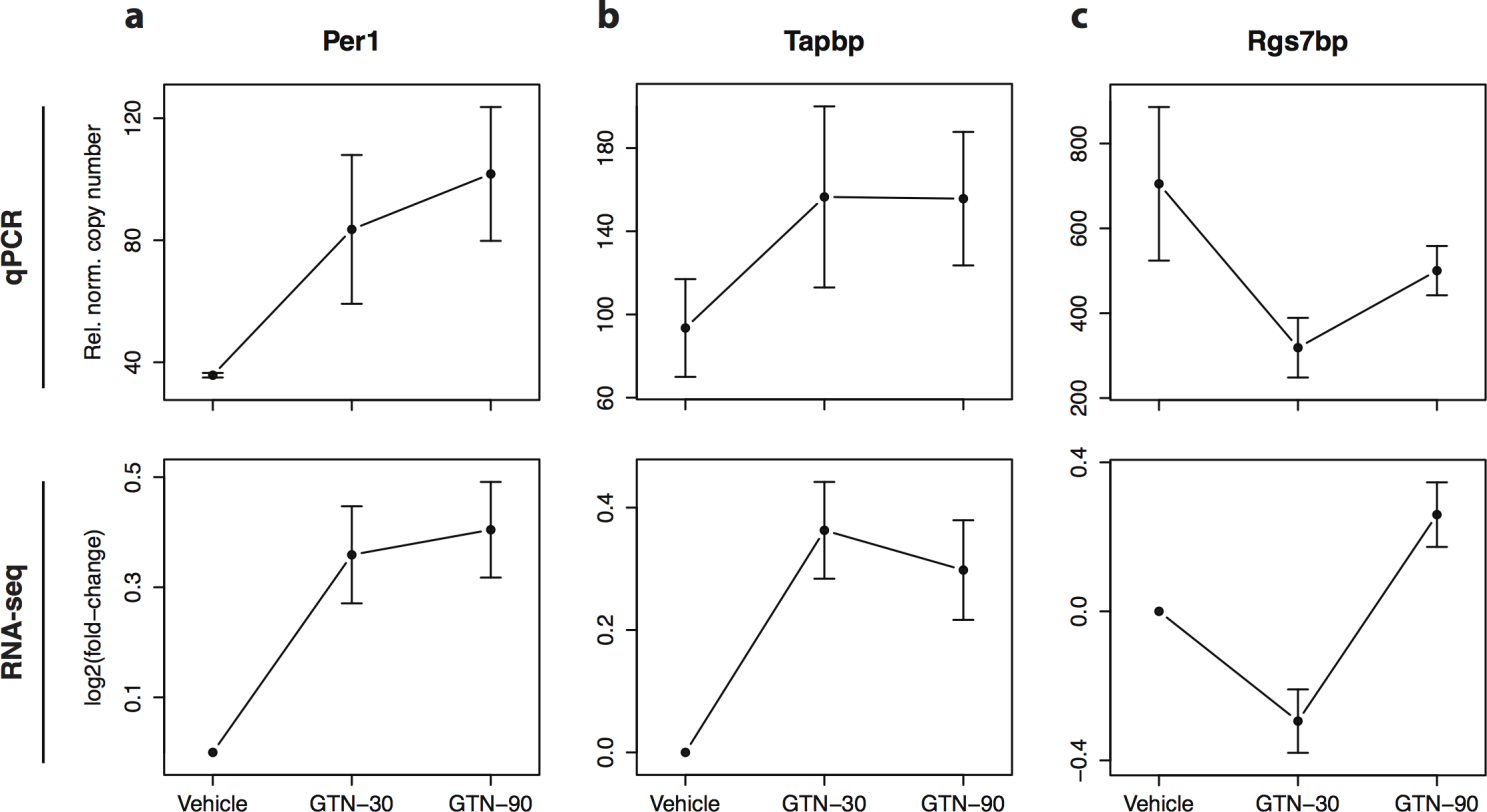
qPCR validation of four differentially expressed genes. Reverse Transcriptase Semi-quantitative PCR (qPCR) analysis on the set of samples used for RNA-seq analysis for four selected differentially expressed genes (A) *RT1-a3* (B) *Per1*, (C) *Tapbp* and, (D) *Rgs7bp*. Upper panels in each subfigure shows the mean and standard error of the relative copy numbers measured by qPCR normalized to *Hprt1* (vehicle n = 2, GTN-30 n = 4, and GTN-90 n = 3). Lower panels show the corresponding log-fold changes estimated using RNA-seq as also shown in Fig 1. Plots with normalized RNA-seq counts for all significant genes are provided in S2 Fig.

The time-dependent dynamics of the gene regulation was divided into four expression patterns: 1) Genes up-regulated after both 30 minutes and 90 minutes (Fig 1, quadrant I) including six genes: apolipoprotein D, *Apod*, glutamine synthetase, *Glul*, period circadian clock 1 gene, *Per1*, TAP binding protein, *Tapbp*, and the RT1 class 1b, locus A3, *RT1*-*A3* gene. These genes are considered having a long-lasting up-regulation in response to GTN, most significant for *RT1-A3* (q = 2.19e-13) as exemplified in the line-plot. 2) Genes with up-regulation at 30 minutes and down-regulation at 90 minutes included only one gene, the 40S ribosomal protein S10, *Rps10* (q = 2.81e-04) (Fig 1, quadrant IV). This gene is considered to respond immediately and transiently to GTN. 3) Genes with a sustained down-regulation at both time points including four genes; membrane bound complement decay-accelerating factor 1, *Daf1*, the enzyme dihydropyrimidinase-related protein 4, *Dpysl4*, the neuron-specific proteinase inhibitor latexin, *Lxn*, and the RT1 class 1b, locus A2, *RT1*-*A2* gene. This pattern of regulation is exemplified by RT1-A2 (q = 1.39e-06) (Fig 1, quadrant III) and these genes are considered to be subject to long lasting suppression in response to GTN. 4) Genes with down-regulation at 30 minutes and up-regulation at 90 minutes, including tripartite motif-containing protein 16, *Trim16*, protein prune homolog 2, *Prune2*, myosin 6, *Myh6*, purinergic receptor P2X, ligand-gated ion channel, 3, *P2x3* and most extremely regulated we find Regulator of G-protein signaling 7-binding protein, *Rgs7bp* (q = 3.09e-07) (Fig 1, quadrant II). Like in quadrant IV, these genes represent a transient response to GTN. For validation of the RNA-seq data results, we selected four significantly differentially expressed genes, *RT1-a3*, *Per1*, *Tapbp* and *Rgs7bp* for qPCR analysis. They all showed time-dependent patterns of regulation similar to the RNA-seq analysis (Fig 2).

### 3.2 GSANOVA analysis

**Fig 3:**
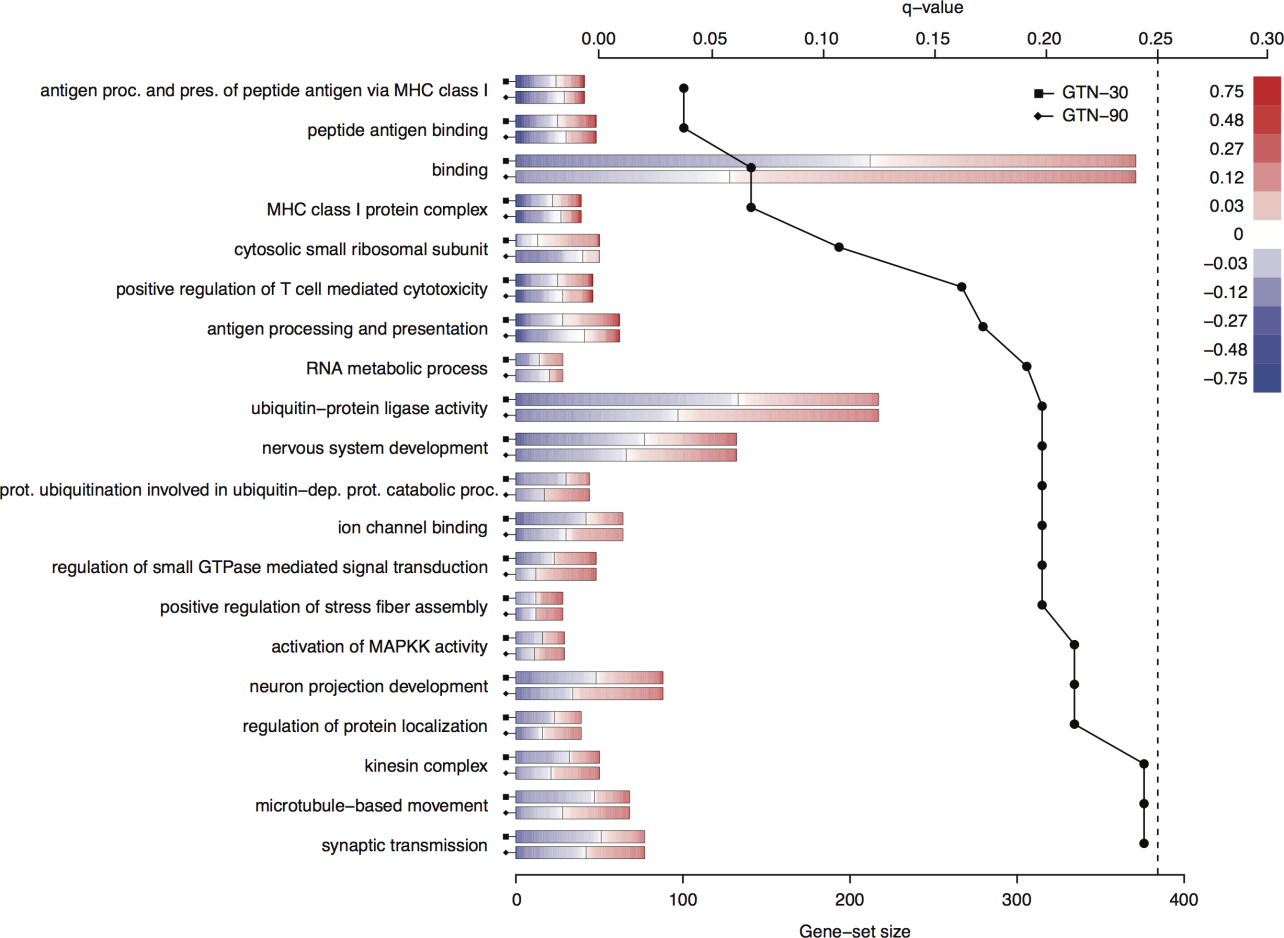
GSANOVA analysis. Enriched gene-sets in the trigeminal ganglia at two time points (30 and 90 minutes) after GTN infusion as determined by GSANOVA analysis. The solid black line shows the q-value for each gene-set (upper axis); gene-sets with q<0.25 (dotted line) were considered significantly enriched. The horizontal bars show the size of each gene-set (lower axis) with the upper and lower bars for each gene-set representing the 30 and 90 minutes time points, respectively. Within each bar, the gene expression change of every gene in the gene-set is shown as a colored vertical line with genes sorted in increasing order of change (blue and red colors represent down- and up-regulation, respectively). Gene expression values were further square root transformed to render the data more uniformly distributed on the color-scale.

We used a newly developed method, Gene Set Analysis Of Variance (GSANOVA), to identify sets of genes that overall exhibit significant changes in response to GTN infusion. This allows us to identify more general functional trends in the data, while simultaneously allowing us to detect changes at the gene-set level that may not have attained statistical significance when looking at individual genes in the set. The gene sets (q < 0.25) shown are enriched in pathways that can be divided into three functions including activation of the major histocompatibility complex (MHC) class I system, plasticity, and signal transduction (Fig 3). The gene expression dynamics of the ten most significant genes in each gene-set are shown as dot-plots in S3 Fig.

The top of the list of most significantly enriched gene sets is dominated by genes involved in MHC class I activation, namely the *Antigen processing and presentation of peptide antigen via MHC class I*, *Peptide antigen binding*, *MHC class I protein complex*, *Positive regulation of T cell mediated cytotoxicity* and *Antigen processing and presentation*. Another cluster of enriched gene-sets is characterized by the involvement of plasticity of cellular structures. These include: *Ubiquitin-protein ligase activity*, *Nervous system development*, *Protein ubiquitination involved in ubiquitin-dependent catabolic process*, *Positive regulation of stress fiber assembly*, *Neuron projection development*, *Regulation of protein localization*, *Kinesin complex* and *Microtubule-based movement*. From the remaining gene sets, signal transduction seems to be profoundly enriched demonstrated by the gene sets *Ion channel binding*, *Regulation of small GTPase mediated signal transduction*, *Activation of MAPKK activity* and *Synaptic transmission*. The detailed gene expression dynamics of the ten most regulated genes in each significantly enriched gene set are shown in S3 Fig.

## Discussion

This is the first study to use RNA-seq to identify differential gene expression patterns with putative involvement in migraine. We infused a dose of GTN that is known to provoke migraine attacks in migraineurs. The same rat model has previously been used to show elevated levels of the neuronal activation marker c-fos in laminae I and II of the spinal trigeminal nucleus caudalis, indicative of trigeminal activation [9]. Here, we used RNA-seq to identify global transcriptional changes in the entire trigeminal ganglion 30 and 90 minutes subsequent to infusion of a low dose of GTN in conscious and freely moving rats.

### 4.1 Transient alterations in gene expression in the trigeminal ganglia in response to GTN infusion

We identified 15 genes that exhibited a significant differential expression in response to GTN-infusion. The results indicate that only six genes are associated with a transient response to GTN after 30 minutes, whereas the majority of altered gene expression display responses sustained for at least 90 minutes. The half-life of GTN is approximately 4 minutes [23], thus a direct effect of NO could be expected within the first 30 minutes after infusion. The gene Rps10 encoding the s10 subunit of the ribosome was the only gene with immediate significant up-regulation followed by normalization within 90 minutes. This could indicate an increase in *de novo* protein synthesis, which is supported by enrichment of the gene-set *Cytosolic small ribosomal subunit* (q = 0.107) revealed by the GSANOVA analysis. *De novo* protein synthesis is most likely reflected by the significant enrichment of gene-sets related to plasticity found 90 minutes after GTN infusion.

### 4.2 Sustained alterations in gene expression in the trigeminal ganglia in response to GTN infusion

#### 4.2.1 Activation of MHC class I genes in response to GTN

In the group of genes showing a sustained and altered regulation at both time points we identified *RT1-a3*, *Tapbp* and, *RT1-A2* along with enrichment of the five gene-sets *Antigen processing and presentation of peptide antigen via MHC class I*, *Peptide antigen binding*, *MHC class I protein complex*, *Positive regulation of T cell mediated cytotoxicity* and *Antigen processing and presentation*. These genes and gene-sets are involved in the MHC class I system, generally a system considered to be a part of the immune surveillance by the adaptive immune system through presenting peptides to cytotoxic CD8^+^ T-cells [24]. The differential expression of these genes was not a consequence of infiltrating T-cells as the expression levels of the T-cell specific genes Cd8a (ENSRNOG00000007178) and Cd8b (ENSRNOG00000007129) as well as Cd3d (ENSRNOG00000015994), Cd3e (ENSRNOG00000016069), and Cd3g (ENSRNOG00000015945) all remained stable regardless of time and GTN-challenging (data can be seen in S1 Dataset).

We also see enrichment in gene sets of ubiquitination (*Ubiquitin-protein ligase activity* and *Protein ubiquitination involved in ubiquitin-dependent catabolic process*) necessary for intracellular protein proteolysis. However, accumulating data indicate that neuronal expression of MHC class I proteins are involved in translation of electrical activity into synaptic plasticity and axonal regeneration in the adult brain [25,26]. In support of these findings is the enrichment of the gene sets *Synaptic transmission* and *Neuron projection development*, respectively.

Increase in c-Fos expression in the spinal trigeminal nucleus caudalis at both mRNA and protein level after GTN stimulation has previously been demonstrated in this experimental model, whereas it was never shown in the trigeminal ganglion [9]. Consistent with this, the Fos-gene is not significantly differentially expressed in response to GTN stimulation in our hands. However, MHC class I molecule expression levels have previously been reported to be up-regulated in the peripheral nervous system in response to neuronal activity [25] and could therefore be interpreted as activation of the trigeminal system in response to a systemic infusion of GTN.

Transcriptional down-regulation of RT1-a2 in the trigeminal ganglion has previously been demonstrated in response to immune stimulation by complete Freund’s adjuvant, whereas a response to the non-immune provocative substance capsaicin was absent [27]. This suggests that GTN provokes a response in the trigeminal ganglia comparable to that of an immune stimulation. All animals included in this study were subjected to the same surgical cannulation and they were randomized according to treatment on experimental days. Thus, the impact of any surgical side effects on the immune system cannot explain these differences. A prominent dural mast cell degranulation and inflammation in response to the same GTN dose has previously been reported and could indicate the provocation of a neurogenic inflammation in the area of trigeminal afferents in the dura mater [12,13]. From human studies, the involvement of pro-inflammatory substances in relation to GTN-induced headache has also been suggested, as migraine patients reported a reduction in headache intensity in the delayed headache phase when given the anti-inflammatory drug prednisolone [28].

#### 4.2.2 Satellite glia cell activation

The trigeminal ganglion has been extensively studied in relation to migraine with a major focus on neurons and neuronal signaling. The neurons are tightly enveloped by satellite glia cells forming distinct anatomical units that offer mechanical and metabolic support but also modulate neuronal function, inflammation, and pain [29–31]. Activation of satellite glial cells has been shown to involve amplification of peripheral inflammatory processes within the ganglia [31,32]. Stimulation with a NO donor caused an immediate release of glutamate from both neuronal and neuronal-astrocytic cell cultures [33,34]. In line with this we find a sustained up-regulation of Glul. This gene is encoding glutamine synthetase enzyme, a catalyst of the conversion of glutamate to glutamine driven by nitrogen sources [35] and indicative of an increased need for glutamate or nitrogen removal within the ganglion after GTN provocation. It is specifically expressed in satellite glia cells and thus suggests an influence of GTN on both satellite glial cells and neurons. This is somewhat speculative as our experimental design was set to capture the activity of the entire ganglion and therefore does not allow for determination of cell-type specific location of transcriptional alterations.

#### 4.2.3 Chronobiological regulation in response to GTN infusion

The period circadian clock 1 gene (Per1) was amongst the most up-regulated genes at both time-points. Per1 is a core gene in the circadian clock system controlling daily physiological and behavioral rhythms [36], and dysfunctional regulation has been linked to impaired quality of sleep [37]. Both migraine and especially cluster headache display a strong chronobiologic component [38–42], and GTN infusion in the human model provokes attacks in both patient groups [5,6]. Tumor necrosis factor-α (TNF-α), a cytokine present in mast cells, induced a mitogen-activated protein kinase (MAPK) and calcium signaling dependent peak in Per1 expression after 30–90 minutes, whereas the homologue genes Per2 and Per3 remained unaffected [43]. This is supported by studies where NO initiated signal transduction through the intracellular MAPK signaling pathway [44,45]. Dural mast cell degranulation has previously been shown in response to systemic GTN-infusion in this model [13] and given that in the present data the gene set “*Activation of MAPKK activity”* is up-regulated, this could be the link between mast cell degranulation, MAPKK activation, and transcriptional alterations in Per1 expression.

#### 4.2.4 Alterations in purinergic receptor expression

Among the genes being most significantly down-regulated at 30 minutes and up-regulated at 90 minutes in response to GTN is the purinergic receptor P2X, ligand-gated ion channel, 3, *P2rx3*. The ionotropic ATP receptor P2rx3 may be responsible for ATP-induced neuronal sensitization [46] and in neuronal cell cultures a rapid and severe ATP depletion was observed in response to NO [34] which could be responsible for our observed initial drop in P2rx3 expression. The neuropeptide CGRP is considered to play a key role in the pathophysiology of migraine [4,47] and was shown to cause a delayed increase in P2rx3 mRNA in cultured mouse trigeminal neurons [48]. Thus, theoretically, the increased expression of P2rx3 receptors in the trigeminal system might be the link between CGRP and NO-induced sensitization.

### 4.3 Conclusion

To the best of our knowledge, this is the first RNA-seq study performed in an experimental animal model with relevance to migraine pathogenesis. We have demonstrated that despite a low number of samples for RNA-seq, we see several indications of GTN-provoked activation of the trigeminal ganglion e.g. a robust activation of the MHC class I system, transcriptional changes in genes known to cause neuroplasticity and changes in signal transduction. Moreover, transcriptional alteration in individual genes like the pain receptor P2rx3 and the circadian clock gene Per1 are interesting findings in relation to NO-provoked headache. Future studies comprising more samples and with the possibility to differentiate between cell-type specific gene alterations could be interesting for a detailed overview of GTN-provoked transcriptional alterations in the trigeminal ganglion.

## Supporting Information

S1 Dataset Sample RNA-seq read counts across Ensembl genes. (TXT)

S1 Fig Outlier detection. Gene read counts were transformed using the regularized logarithm function in the DESeq2 R package to adjust for sequencing depth and to render the data homoscedastic. Shown are pairwise euclidean distances between samples along with the corresponding hierarchical clustering dendrograms (**a**). Sample TG-90-4 was identified as a suspected outlier and excluded from further analysis. The analysis was repeated after exclusion of the outlier and confirmed that there were no further outliers (**b**).
(TIFF)

S2 Fig Normalized RNA-seq read counts for significant genes. Normalized RNA-seq read counts for the 15 genes that exhibited statistically significant changes in response to GTN treatment. The Y-axis is logarithmic. Raw counts for all genes are provided as S1 Dataset.
(TIFF)

S3 Fig Gene set expression dynamics. 20 gene-sets were enriched for transcriptional responses to GTN as determined using GSANOVA (q < 0.25). Each 2D-plot shows the expression dynamics of the ten most significant genes in the corresponding gene-set as determined using likelihood-ratio tests. Dots indicate log2(fold-changes) with the X- and Y-dimensions representing the responses at 30 and 90 minutes, respectively. The horizontal and vertical lines show the standard error of the log2(fold changes) after 30 and 90 minutes, respectively.
(TIFF)

S1 Table Estimated log2 fold-changes and likelihood ratio test q-values for significant genes. (XLSX)
