# Supplementary figures and images for: RNA Sequencing of Trigeminal Ganglia in Rattus Norvegicus after Glyceryl Trinitrate Infusion with Relevance to Migraine
Source: PLoS One. 2016 May 23;11(5):e0155039. doi: 10.1371/journal.pone.0155039 (PMC4877077; doi:10.1371/journal.pone.0155039)

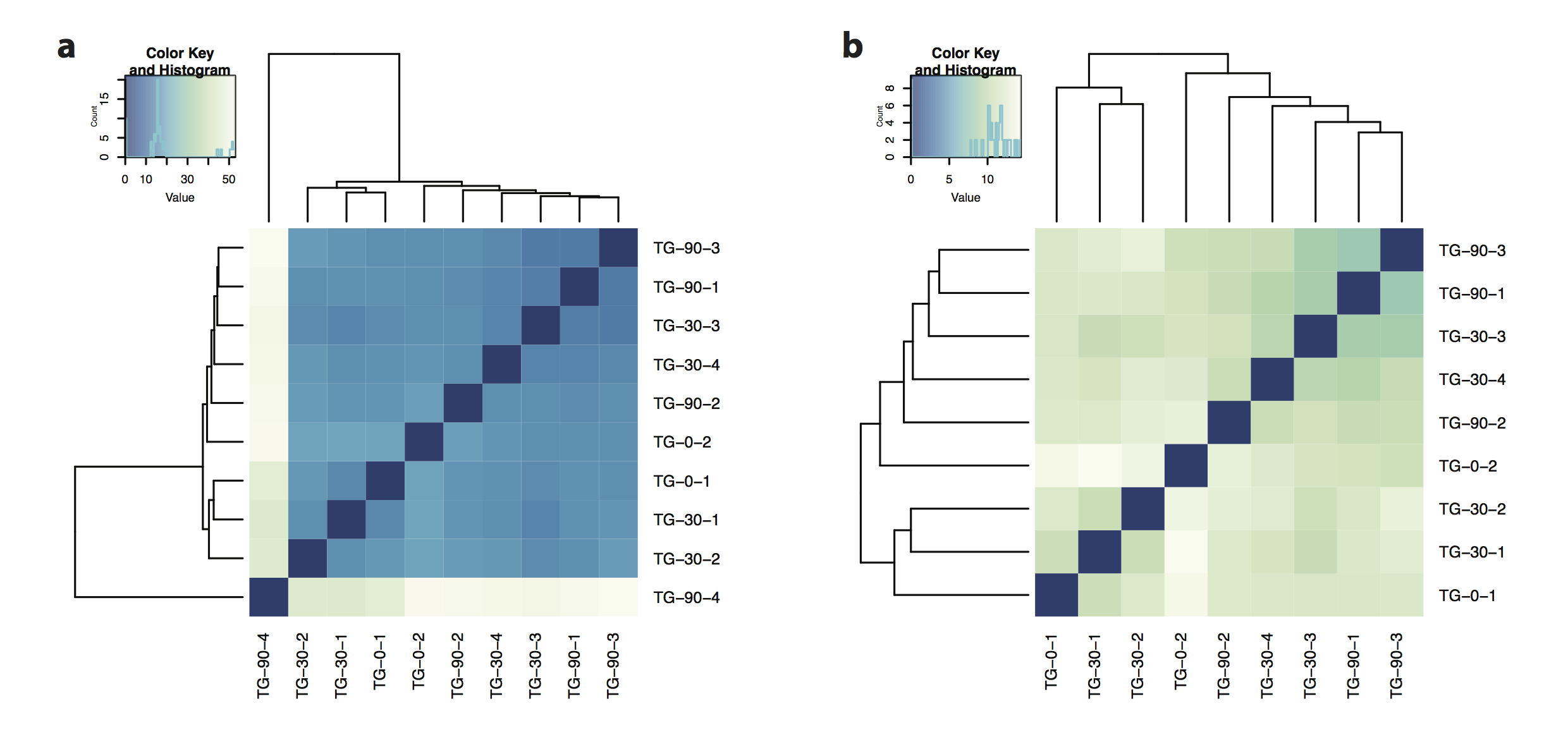

Supplement: S1 Fig — Gene read counts were transformed using the regularized logarithm function in the DESeq2 R package to adjust for sequencing depth and to render the data homoscedastic. Shown are pairwise euclidean distances between samples along with the corresponding hierarchical clustering dendrograms (a). Sample TG-90-4 was identified as a suspected outlier and excluded from further analysis. The analysis was repeated after exclusion of the outlier and confirmed that there were no further outliers (b). (TIFF) [file pone.0155039.s002.tiff]

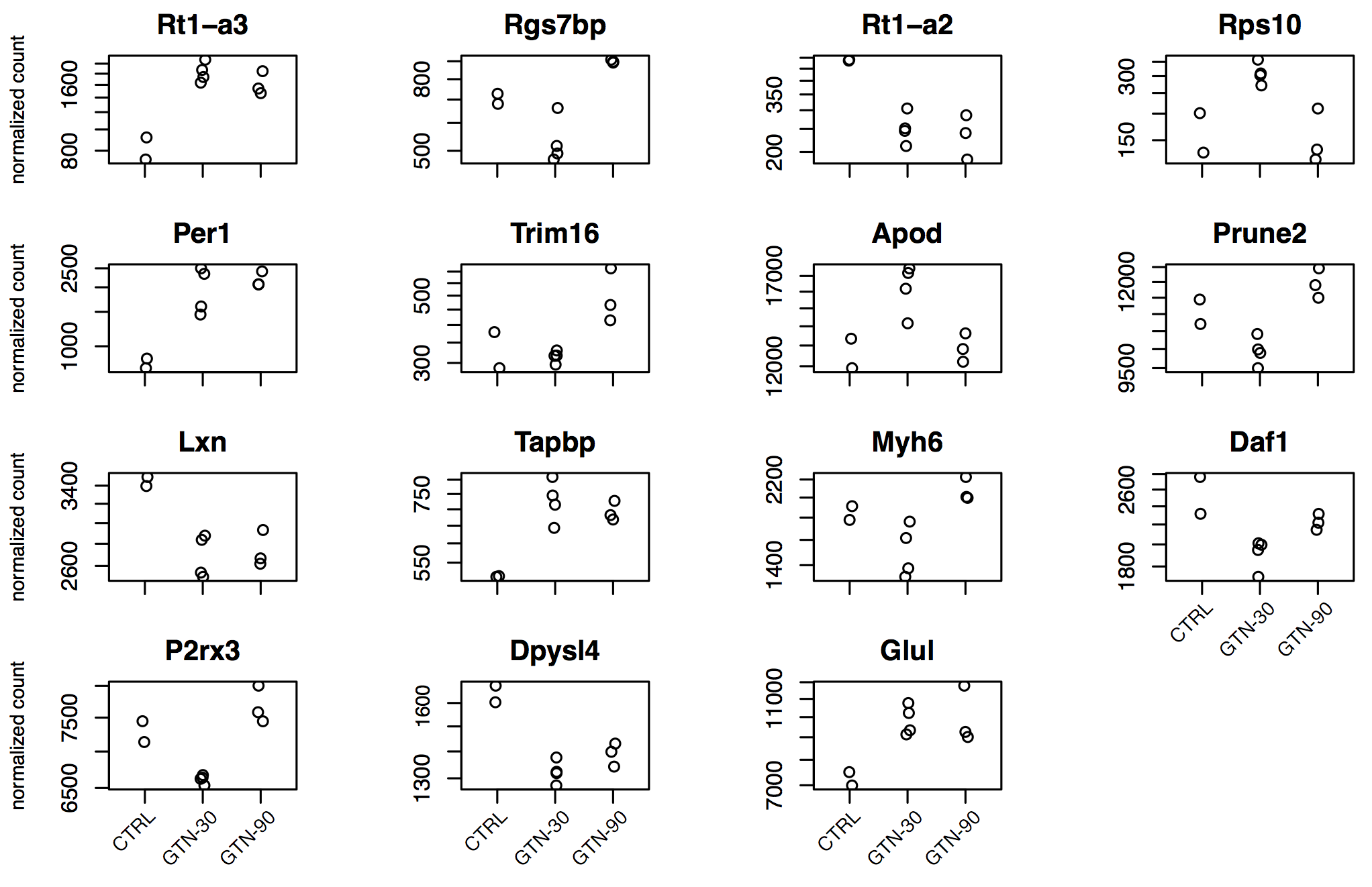

Supplement: S2 Fig — Normalized RNA-seq read counts for the 15 genes that exhibited statistically significant changes in response to GTN treatment. The Y-axis is logarithmic. Raw counts for all genes are provided as S1 Dataset. (TIFF) [file pone.0155039.s003.tiff]

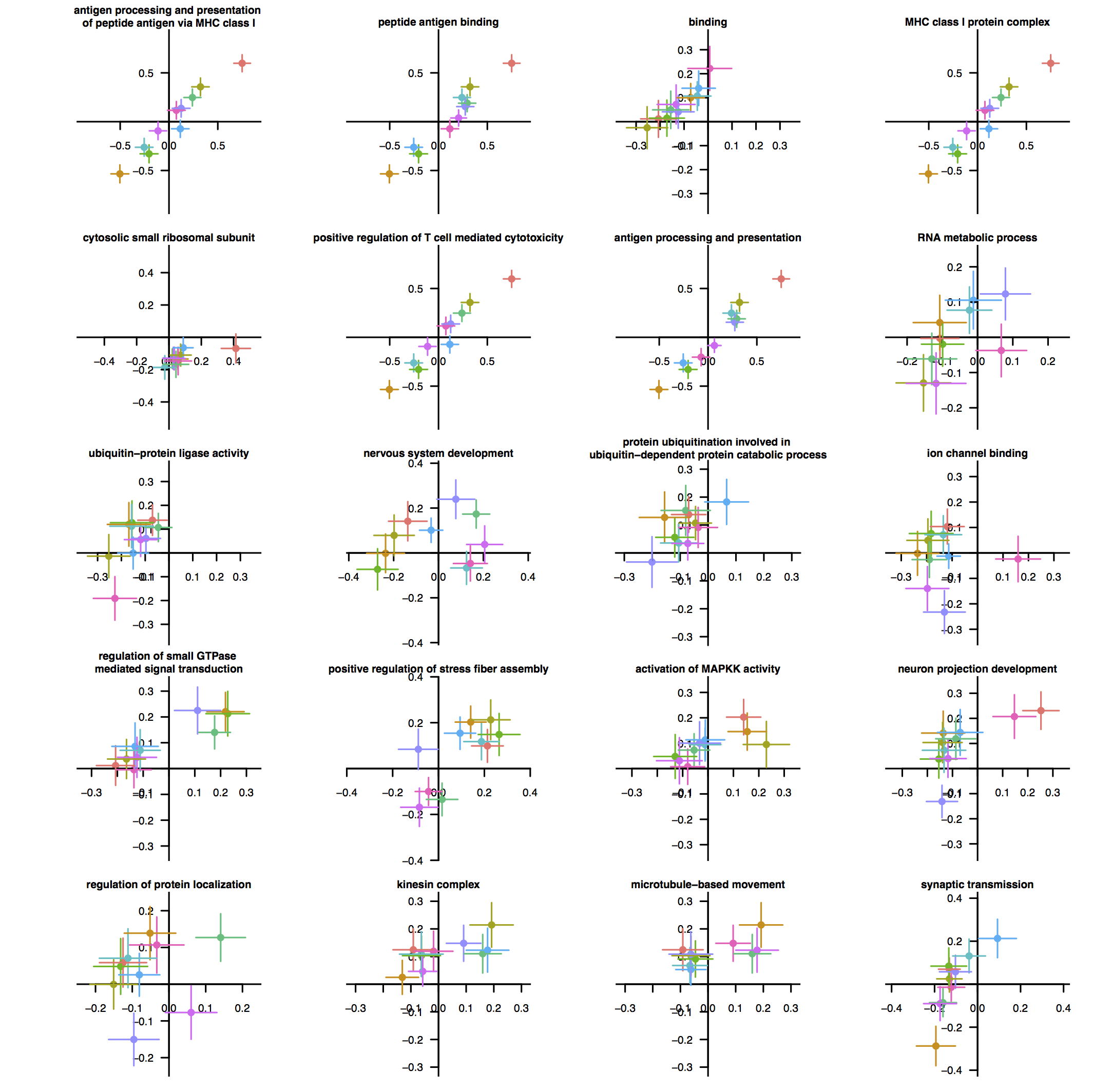

Supplement: S3 Fig — 20 gene-sets were enriched for transcriptional responses to GTN as determined using GSANOVA (q < 0.25). Each 2D-plot shows the expression dynamics of the ten most significant genes in the corresponding gene-set as determined using likelihood-ratio tests. Dots indicate log2(fold-changes) with the X- and Y-dimensions representing the responses at 30 and 90 minutes, respectively. The horizontal and vertical lines show the standard error of the log2(fold changes) after 30 and 90 minutes, respectively. (TIFF) [file pone.0155039.s004.tiff]
